# Quality of life in idiopathic dystonia: a systematic review

**DOI:** 10.1007/s00415-018-9119-x

**Published:** 2018-11-20

**Authors:** Ayesha Girach, Ana Vinagre Aragon, Panagiotis Zis

**Affiliations:** 1 Sheffield Institute for Translational Neuroscience, University of Sheffield, Sheffield, UK; 2 Academic Department of Neurosciences, Sheffield Teaching Hospitals, NHS Foundation Trust, Sheffield, UK; 3 Medical School, University of Cyprus, Nicosia, Cyprus

**Keywords:** QoL, CD, BEB, BTX, DBS

## Abstract

**Objective:**

Dystonia is characterised by sustained muscular contractions frequently producing repetitive, twisting and patterned movements. The primary aim of this systematic review was to establish how quality of life (QoL) is affected in idiopathic focal, multifocal and segmental dystonia. This review aimed to evaluate variations in QoL between different subtypes of dystonia, identify the determinants of QoL and assess the effects of different treatments on QoL.

**Methodology:**

A systematic computer-based literature search was conducted using the PubMed database to search for papers on QoL in idiopathic focal, segmental, multifocal and generalized dystonia. We identified 75 studies meeting our inclusion criteria. Information was extracted regarding prevalence, demographics and response to treatment where indicated.

**Results:**

This review revealed QoL to be a significant yet often overlooked issue in idiopathic dystonia. Data consistently showed that dystonia has a negative effect on QoL in patients compared to healthy controls, when measured using disease-specific and generic QoL measures. The majority of studies (*n* = 25) involved patients with cervical dystonia, followed by benign-essential blepharospasm (*n* = 10). Along with the beneficial effect to the dystonia symptoms, treatment using Botulinum Toxin and Deep Brain Stimulation is also effective in improving overall QoL across the majority of subtypes.

**Conclusion:**

The findings demonstrate that patients’ QoL should routinely be assessed and monitored, as this may affect subsequent management. Further research will allow for more robust management of factors contributing to impaired QoL, aside from the physical defects found in dystonia.

## Introduction

Dystonia is a neurological disorder characterised by continuous or intermittent muscular contractions, causing abnormal, often repetitive involuntary movements, postures, or both [1]. Similar to other movement disorders, it is a highly stigmatizing and disabling condition. Dystonia can be divided according to its bodily distribution: focal (localised to a single body region), segmental (spread to contiguous parts of the body), multifocal (spread to non-contiguous parts of the body) and generalized [1, 2].

Several studies have suggested that quality of life (QoL) in dystonia is not exclusively determined by the presence of the well-defined motor symptoms, but by other clinical, demographic, social and psychological factors [3–5]. Boundaries of dystonia and psychiatry are still not well established, as an excess of psychopathology has been reported in dystonic patients [6]. However, there is conflicting evidence as to whether these are secondary to motor causes and medications or whether these are symptoms of an underlying primary defect in neuronal processing and neurochemical defects.

The aim of this study was to systematically review the current literature regarding QoL in patients with idiopathic focal, segmental, multifocal and generalized dystonia. We aimed to evaluate variations in QoL between the different subtypes, and more specifically identify sociodemographic variables and clinical symptoms having an effect on patients’ QoL. Additionally, we aimed to identify what the effects of different treatments are on QoL. To our knowledge this is the first systematic review on the topic.

## Methodology

### Protocol

This review was not registered on a public database. It has been approved and registered on the database of dissertation projects for the MSc in Clinical Neurology at the University of Sheffield.

### Search Strategy

A systematic computer-based literature search was conducted on 19th February, 2018, using the PubMed database, covering all articles published in the date range 01/01/1990-19/02/18. For the search we used two Medical Subject Headings (MeSH) terms in title or abstract. Term A was “dystonia” or “dystonic” or “anterocollis” or “torticollis” or “laterocollis” or “blepharospasm” or “writer’s cramp”. Term B was “quality of life” OR “qol”. Filters were species to humans, language to English and full text to be available. Additionally, we pursued the reference lists of the identified papers to try and include further papers reporting aspects of QoL in idiopathic dystonia populations that were not revealed based on the above mentioned search.

### Inclusion and exclusion criteria

In order to be included in the review articles were required to meet the following criteria: (1) be original articles; (2) study human subjects; (3) be written in English; (4) refer to idiopathic dystonia; (5) refer to the QoL of subjects. The exclusion criteria for the articles were as follows: (1) be book chapters, reviews, meta-analyses, development of clinical scales, letters to the editor and editorials not providing new data, study protocols; (2) be articles on patients with non-idiopathic dystonia; (3) be articles with a lack of individual results for idiopathic focal dystonia/ segmental dystonia/ multifocal dystonia/ generalized dystonia even if these subjects were included in the study; (4) be articles which did not explore QoL as an outcome measure.

### Synthesis of results

This study is reported in accordance with the ‘Preferred Reporting Items for Systematic Reviews and Meta-Analysis (PRISMA) guidelines’ [7]. A database was developed using the Statistical Package for Social Science, version 23 for Mackintosh. Pooled frequencies and descriptive characteristic of demographic parameters (age and gender) for each type of dystonia were calculated.

### Assessment of bias

The studies included in this review did not contain sufficient quantitative data for which risk bias tools such as funnel plots could be conducted. A risk of bias tool was, therefore, not used.

### Compliance with ethical guidelines

This article is based upon previously published studies. The article is in compliance with the journal’s ethical guidelines.

## Results

### Selected studies

**Fig. 1:**
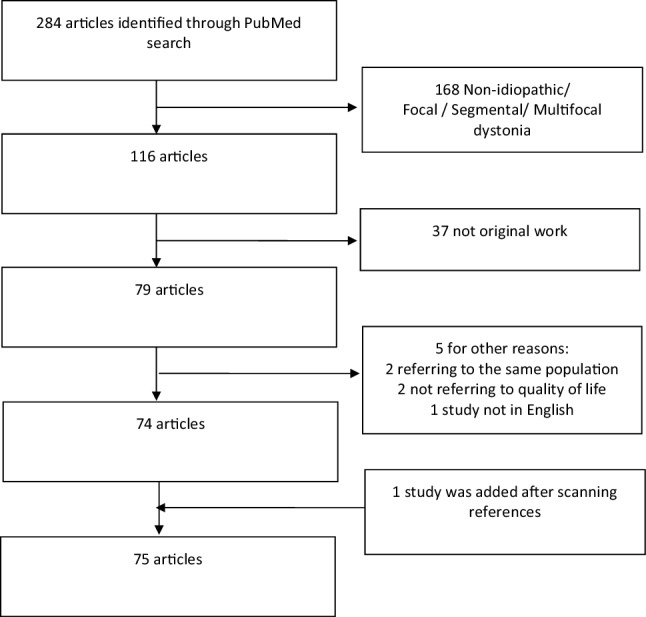
PRISMA chart displaying article selection process

A PRISMA chart displays the process of article selection (Fig. 1). The PubMed search identified 284 articles. A total of 210 articles were excluded during the eligibility assessment, and a further one article was added after scanning the references of the included studies. In total, 75 articles met the inclusion criteria. Case series constituted the commonest type of paper (65.3%) included in this review. More than 50% of articles reporting QoL in dystonia have been published in the past 8 years. Twenty-five articles analysed cervical dystonia (CD) whilst only one article reviewed QoL in musician’s dystonia. A summary of trial and patient characteristics has been demonstrated in Table 1.

**Table 1:** Summary of trial and patient characteristics

*Total number of papers included in this review*	75
*Type of paper (%)*	
Case report	4 (5.3)
Case series	49 (65.3)
Case-controlled study	13 (17.3)
Randomized controlled trial	7 (9.3)
Pharmaco-economic	2 (2.7)
*Total number of patients*	6,528
*Mean number of patients, per paper (SD)*	87.0 (145.5)
*Mean age of dystonia onset, in years*	47.6
*Male to female ratio*	1:2
*Type of dystonia studied, number of papers (%)*	
Blepharospasm	10 (13.3)
Cervical	25 (33.3)
Laryngeal	4 (5.3)
Musician’s	1 (1.3)
Oromandibular	3 (4.0)
Segmental	3 (4.0)
Generalized	3 (4.0)
Various types	26 (34.7)
*Number of publications per decade*	
Until 2000	4 (5.3)
2000–2009	33 (44.0)
2010–2018	38 (50.7)

### Patient demographics

A total of 6528 participants were included in this review. Participants of individual trials were all equal to or above the age of 16. The mean age of onset of dystonia was 47.6 years; however, there were variations in this according to the type of dystonia. Musician’s dystonia had the lowest mean age of onset at 34.6 years, whilst the highest mean age of onset at 52 years was found in oromandibular dystonia (OMD) patients. Overall, females represented double the number of participants compared to males. Most types of dystonia had more female patients, except segmental dystonia in which more male patients were identified and  generalized idiopathic dystonia where the male to female ratio was 1:1. The main participant demographics per dystonia subtype are demonstrated in Table 2.

**Table 2:** Summary of patient characteristics

Type of dystonia	Number of papers	Number of patients	Male:female	Mean age of dystonia onset
Blepharospasm	10	378	2:5	46.9 years
Cervical	25	2771	1:2	45.7 years
Laryngeal	4	75	1:4	N/A
Musician’s	1	243	2:7	34.6 years
Oromandibular	3	72	1:2	52.0 years
Segmental	3	42	4:3	45.9 years
Generalized	3	49	1:1	N/A

### Assessment of QoL

In total, 21 different tools were used to assess QoL in dystonic patients. More than half of the studies (52.0%) utilised the SF-36 tool with the second commonest being the EQ-5D (12.0%), both of which are generic health status measurements. A number of disease-specific questionnaires were employed, most notably the CDQ-24 (*n* = 6) for the assessment of cranio-cervical dystonia, constituting the third commonest questionnaire used [8, 9]. A tabulated breakdown of the named questionnaires included in this review are provided in Table 3.

**Table 3:** Questionnaires used to assess QoL

Questionnaire	*N* (%)^a^
30-item Hemifacial Spasm Quality of Life (HSF-30)	1 (1.3)
7-item Hemifacial Spasm Quality of Life (HFS-7 QOL)	1 (1.3)
Cervical Dystonia Impact Profile-58 (CDIP-58)	4 (5.3)
Clinical Global Impression Scale	2 (2.7)
Craniocervical dystonia questionnaire (CDQ24)	6 (8.0)
Dry Eye-Related Quality of Life Score (DEQS)	1 (1.3)
EQ-5D	9 (12.0)
Fugl-Meyer questionnaire	1 (1.3)
Glasgow Benefit Inventory	4 (5.3)
Nottingham Health Profile (NHP)	2 (2.7)
Oromandibular dystonia questionnaire (OMDQ-25)	2 (2.7)
PDQ-39	1 (1.3)
Questionnaire— not specified	3 (4.0)
Questionnaire on Life Satisfaction	1 (1.3)
SF-20	1 (1.3)
SF-36	39 (52.0)
Spitzer Quality of Life Index (SQLI)	1 (1.3)
Visual Function Questionnaire (VFQ-25)	2 (2.7)
Voice Handicap Index (VHI)	2 (2.7)
Voice-Related Quality of Life (V-RQOL)	2 (2.7)
WHOQOL-BREF	1 (1.3)

^a^A number of papers used more than one questionnaire

### Cervical Dystonia

CD is the commonest type of focal dystonia addressed in this review. The Cervical Dystonia Patient Registry for Observation of Onabotulinumtoxin A Efficacy (CD PROBE) is the largest observational study (n = 2771) in CD [10].

#### Determinants of QoL in CD

##### Age

There was found to be no association between age at onset and QoL; however, age at assessment had a negative correlation with the physical function domain of SF-36 in CD [11]. This supports findings from Camfield et al. and Slawek et al. where a correlation exists between increasing age and decreasing QoL (role-physical) [4, 12].

##### Disease severity

It has been reported that higher scores in Toronto Western Spasmodic Torticollis Rating Scale (TWSTRS), thus more severe cases of CD, are associated with lower scores in social functioning sub-domain of SF-36 [13, 14]. However, two studies prior to this found that there is no correlation between severity of CD and low QoL, though in both these studies the Tsui scale was used as opposed to TWSTRS. Therefore, current evidence is contradictory as to whether disease severity affects the extent to which QoL is impacted; however, the fact that different tools were used may explain the differences in these results.

##### Comorbidity with mood disorders

The main predictors of poor QoL in CD has consistently been found to be a poor ‘body concept’, the feeling of a physical defect and the subsequent depression associated with this [3, 4, 15, 16]. Depression and anxiety have been found to be major factors influencing QoL in dystonic patients, as well as other psychological issues such as low self-esteem, embarrassment and limited social interaction that often accompanies dystonia [3, 4, 17–19]. This highlights the relationship between dystonia and psychiatric illnesses to be bidirectional and dynamic.

#### QoL aspects in CD

##### Psychiatric features

It has been identified that the extent of dystonia and body parts affected have significant impact on depression; those with head or neck involvement reported the worst outcomes in QoL [4, 11, 20–22]. However, this could be explained by CD being the most associated with immediately visible postural abnormality, and thus the strongest correlation with social stigmatization and embarrassment. An implication of this clinically is that dystonia patients may benefit from cognitive behavioural therapy, specifically addressing the patients negative body concept [20].

##### Pain

In the CD PROBE study, pain correlated with the perceived severity of CD based on the TWSTRS score and physician [10]. This indicates that pain correlates with disease severity, but this relationship is complex as it is not clear whether pain directly contributes to an increase in perceived severity or if pain arises as a result from increased severity [5, 10, 23].

##### Impact on work and employment

The CD PROBE study showed moderate/severe pain had the greatest impact on employment status as these patients were four times more likely to be unemployed as opposed to those with no/mild pain, demonstrating the detrimental effects of dystonia on occupational functioning [10]. By design, registry studies are not blinded or randomized and lack control groups for comparison, highlighting a limitation to the study.

##### Fatigue

Fatigue and sleep disturbances were demonstrated as common and significant issues in patients with dystonia [24]. The exact pathophysiology underpinning fatigue in dystonia is poorly understood; however, one theory is that increased energy demands from excessive muscle contractions cause fatigue.

##### Sleep disturbances

Eichenseer et al. conducted the largest case controlled study to date, studying sleep quality in CD. Limitations included the lack of a control group, and a single visit study. The implications of this clinically are that a separate focus for screening and managing sleep disturbance in dystonia is recommended [25].

#### Effect of treatment on QoL in CD

##### Botox in CD

Botulinum toxin (BTX) A treatment has become a widely used treatment for CD, as it has proven both safe and effective, with longer treatment regimens associating with better SF-36 scores [10, 12, 26–29]. Sethi et al. reported satisfaction with BTX treatment was dependent on the timing of assessment with regards to the injection cycle. Satisfaction was highest at the time of peak therapeutic effect and lowest immediately prior to the next injection [30]. Huang et al. conducted a case controlled study using an ultrasound-guided local injection of BTX A combined with an orthopaedic brace significantly reduces muscle tension and improves QoL [31]. A common type of pain reported by patients with CD is headache [23]. It has been found that BTX A is an effective treatment for headache in patients with CD; however, larger randomized controlled trials are warranted [32].

There is a growing awareness that a significant proportion of CD patients discontinue therapy. This could be considered a reason for reduce QoL in dystonic patients, as there is dissatisfaction with their treatment. Further efforts to optimize administration of BTX are needed, such as reducing side effects or adding on therapies [33].

##### BTX combined with physical therapy

In addition to BTX, physical therapy can be added to the treatment to achieve better results. Queiroz et al. assessed in a case controlled study whether BTX in addition to physical therapy had significant benefits to overall QoL [34]. Zetterberg et al. also included manual muscle strengthening in a treatment protocol, and improvements in QoL were found in the majority of patients [35].

##### Deep brain stimulation of globus pallidus internus in CD

There is substantial variability in the responsiveness to Deep Brain Stimulation (DBS), most likely due to the variability in causes of dystonia; however, bilateral pallidal stimulation continues to be a highly effective and safe treatment option for patients, with reduction in disability and overall improved QoL lasting for up to 5 years [36–39]. Differences in methodological approach, surgical technique, programming regimes and clinical presentations make determining outcome predictors in these subgroups more challenging. With regard to QoL, the physical and mental health scores show significant improvements [36–38]. Well designed, randomised controlled trials are needed to validate the theoretical advantages of constant current DBS over constant voltage DBS [40].

##### DBS of subthalamic nucleus in CD

Few studies have investigated QoL in CD patients undergoing Subthalamic Nucleus (STN) stimulation [41, 42]. Patients in a study by Ostrem et al. who were treated with STN stimulation found significant improvements in physical health, but not mental health, whilst 90% of patients in this case series showed long-term tolerability and sustained improvements in QoL and disability [43]. The first randomized double-blind study attempted to compare the STN as a target for DBS as opposed to the GPi and found there to be no difference in stimulation of either, with regards to the effect on QoL [44].

##### Peripheral denervation in CD

Peripheral denervation is a procedure attempting to cut the nerve supply to the muscles causing the most prominent dystonic movements. Münchau et al. found selective peripheral denervation to be a treatment option for those with secondary, but not primary BTX treatment failure [45]. It was found reinnervation of muscles was common and compromised outcomes, despite there being significant improvements in many aspects of practical daily living in the short term. An improvement in functional outcome does not imply a better QoL; thus more recently, a study conducted a long-term follow-up of 61 procedures with selective peripheral denervation, assessing QoL as an outcome [46]. The patients rated their QoL as significantly better postoperatively in general, although specific domains of the Fugl-Meyer scale were not significantly changed at long-term follow-up. The Fugl-Meyer scale is a generic scale and may have failed at identifying aspects relevant to CD patients.

### Blepharospasm

Our results found 10 papers addressing QoL in benign essential blepharospasm (BEB), with a total of 378 patients, forming the second largest group of patients in this study.

#### Determinants of QoL in BEB

##### Disease duration

Muller et al. found those with a longer duration of BEB correlated with a better QoL [11]. Justification for this is based on the idea that patients with longer term illness adapt to their physical disabilities, and adjust their ‘norms’, so negative effects of the disease are not perceived to such a great extent.

##### Gender

In their study, Muller et al. reported that women with BEB show significantly worse scores in 5 out of 8 domains on the SF-36 compared to male patients [11]. This was supported by Zhang et al. who reported the results indicated being female was a determinant of poor QoL besides depression, anxiety and functional disability [47]. This could be explained by the fact that women are more sensitive to disfigurement.

##### Cognitive impairment

A case controlled study by Yang et al. found that patients with cognitive deficits in BEB had poorer QoL especially in the domains physical role, physical functioning and social functioning [48]. An important factor helping patients cope with disease is knowledge; those with a higher educational level predicted a higher knowledge of their condition and in turn a better improvement in QoL after BTX treatment [49].

##### Psychiatric features

A number of studies have described associations between BEB and psychiatric problems, including depression and obsessive–compulsive disorder [11, 50–52]. Hall et al. employed a control group with hemifacial spasm, to determine the extent to which mental health problems exist at higher rates in patients with BEB. The use of patients with hemifacial spasm as a reference group provides an interesting comparison, given the overlapping clinical profiles of both movement disorders. Those with BEB experienced a greater reduction in vision-targeted health-related QoL and were more prone to depression and anxiety [50].

#### Effect of treatment on QoL in BEB

##### BTX in BEB

Many reports have demonstrated an improvement in QoL in BEB patients with BTX injections [11, 51, 53–57]. However, limited studies have assessed QoL during long-term injection regimes [50, 58]. A number of studies demonstrated long-term benefits to BTX [52, 59]. Early introduction of BTX is recommended as there are added benefits to the patient and family. The patient can return to work, and their improved social interaction leads to less depression [59]. Additionally, the patient has added security in their everyday life, as the possibility of personal injury is reduced, in turn reducing the burden on family members [52]. A case controlled study found no link between the combined use of alleviating maneuvres (touching specific areas of the face, i.e the upper eyelid) and BTX treatment with better QoL outcomes [60].

### Laryngeal dystonia

Adductor spasmodic dysphonia is a type of focal laryngeal dystonia with great implications on QoL [61]. Untreated patients have a very low voice-related quality of life (V-RQoL) according to the voice handicap index [62]. BTX is considered the treatment of choice in laryngeal dystonia; however, disadvantages include changes in the pitch or volume of the voice and a high drug cost [63–65].

### Focal hand dystonia: writer’s cramp and musician’s dystonia

Lee et al. (*n* = 243) demonstrated in a case controlled study that patients suffering with focal hand dystonia are not less satisfied with their jobs or life in general than unaffected musicians [66]. This disproved the study’s hypotheses and supports the subjective satisfaction paradox, describing the phenomenon that adverse events in an individual’s life have little impact on an overall satisfaction with life as long as a minimum of existential needs are secured. The findings from this study highlight that clinicians should discuss with patients the importance of maintaining a healthy mindset and the high probability of coping with the situation irrespective of the course of the disease [66]. Pekmezovic et al. were the first to address QoL in focal hand dystonia patients [18]. This population scored better in all SF-36 domains except role-functioning when compared with BEB and CD. This could be explained by patients having a younger age of onset, a higher level of education, more frequently employed and less pain observed than CD or BEB patients.

### Lingual and oromandibular dystonia

Lingual dystonia (LD) often co-exists with OMD and rarely exists as isolated LD alone. There is limited literature regarding the efficacy of BTX for isolated LD in larger case series, in particular its association with patient QoL. Nastasi et al. proved BTX to have a positive change in both mixed phenotypes (a combination of OMD and LD subtypes) and in isolated LD, with regards to OMDQ-25 scores [67]. The lowest change was observed in the functional eating category, suggesting this complex movement is the least susceptible to a satisfactory response from BTX. The results showed those with an additional jaw deviation pattern had an increased QoL outcome post-treatment, whereas those with associated jaw tremor had poorer QoL outcomes.

Limited literature exists regarding QoL in the isolated OMD population. Only one study focused on QoL in patients after BTX treatment. Patients felt more optimistic, less embarrassed and more social post-treatment. However, the retrospective nature and self-selected survey may have represented a skewed population [68].

### Segmental dystonia

Basurovic et al. found the most important predictors of poor QoL in patients with segmental dystonia were disease severity, low acceptance of illness, depression and low self-esteem [69]. The efficacy of BTX in segmental dystonia is restricted due to dose limitations [70]. Reports on the effectiveness of DBS in segmental dystonia are limited [70–72]. Bilateral GPi DBS led to significant improvements in QoL according to Mueller et al. and a sustained improvement of motor symptoms by 60% in the 18-month follow-up according to Blahak et al. [70, 73]. Importantly, studies reporting on segmental dystonia fail to clarify the specific type studied, and due to the heterogeneity of this category, it is difficult to make generalisations and comparisons.

Adult onset axial dystonia accounts for 10% of segmental dystonia. Patients develop a negative personal image, depression and social isolation. A study showed that despite the refractory nature of the disease, there was an excellent response to GPi DBS and a significant improvement in QoL. The implications of this clinically are that DBS should be considered early in the disease course, considering the poor response of adult onset axial dystonia to medical treatment and the significant impact on QoL [74].

### Generalized dystonia

Although generalized dystonia is in the majority of cases genetic, thus not idiopathic, there are cases that remain idiopathic. Literature on QoL in idiopathic generalized dystonia is limited; however, our literature search identified 3 studies evaluating the effect of deep brain stimulation on QoL of patients with idiopathic  generalized dystonia. Jahanshai et al. found that when patients were treated with pallidal DBS there was no significant alteration in depression, apathy or anxiety, despite the improvement in the severity of disease with the intervention [75]. However, this result was somewhat in contrast to the findings of the study conducted by Vidailhet et al. [76], where patients treated with pallidal DBS were found to have improvements in QoL closely related to their motor score improvements; however, an absence of beneficial effect in the social functioning and role-emotional of the SF36 could be attributed to psychological factors such as self-esteem [76]. Cao et al. looked at the effects of STN DBS and found a steep improvement in QoL in the first month of treatment [77]. Improvements in QoL were evident up until 3 years; however, subsequently the improvement plateaued.

### Pharmaco-economic studies and the burden of poor QoL

Our literature search identified two papers discussing the pharmaco-economic impact of BTX treatment on QoL [78, 79]. Measuring QoL is important in assessing cost-effectiveness and allocating resources where funding is limited. Both studies are over 18 years old, and considered largely out of date; however, the conclusions drawn from the studies are relevant today. Significant benefits of BTX were observed both in QoL and clinically, outweighing the cost of BTX. However, both these studies were open-label and, therefore. may overestimate the effect of BTX on QoL.

## Conclusions

This systematic review has identified the following key points:

- Research into QoL in dystonia is increasing. The increasing research interest reflects in part the advancement in understanding QoL is an equally important endpoint for patients with dystonia as objective outcomes.
- Implications of this clinically, are these elements affecting QoL may help us refine our treatment options and reorient our treatment goals.
- Overall findings suggest patients with any form of dystonia have a reduced QoL compared to healthy controls. The reasons underpinning this vary not exclusively to the clinical characteristics of the dystonia, but to other factors such as social and demographic variables, and non-motor symptoms. Inherently, the stigma attached to dystonia requires significant social adaptation and coping mechanisms by patients, in turn impacting on QoL. This calls for further clinical research in the field as well as regular routine monitoring and assessment of QoL in dystonic patients.
- The review highlighted the majority of studies in the literature focus on QoL in CD and BEB, reflecting the respective higher prevalence of these subtypes. This is a prerequisite for more research looking into QoL in the other subtypes of dystonia.

## Limitations

- Outcome measures such as the SF-36 scale are self-reported questionnaires; thus there is potential for an inherent response bias. This questions whether or not the QoL scales are really reflecting the true nature of the QoL of patients who have dystonia.
- A more comprehensive search using other databases, rather than PubMed alone, may have produced a greater number of articles suitable for final analysis.
- Studies should not be excluded due to language, rather they should be professionally translated, which was not possible in this case due to financial and time constraints.
